# Mini Review: Forensic Value of Aquaporines

**DOI:** 10.3389/fmed.2021.793140

**Published:** 2021-12-17

**Authors:** Julian Prangenberg, Elke Doberentz, Burkhard Madea

**Affiliations:** Institute of Legal Medicine, University Hospital Bonn, Bonn, Germany

**Keywords:** aquaporins (AQPs), immunohistochemistry, wound vitality, drowning, SIDS

## Abstract

Forensic pathologists are routinely confronted with unclear causes of death or findings. In some scenarios, it can be difficult to answer the specific questions posed by criminal investigators or prosecutors. Such scenarios may include questions about wound vitality or causes of death when typical or landmark findings are difficult to find. In addition to the usual subsequent examinations to clarify unclear causes of death or special questions, immunohistochemical analysis has become increasingly important since its establishment in the early 40s of the 20th century. Since then, numerous studies have been conducted to determine the usefulness and significance of immunohistochemical investigations on various structures and proteins. These proteins include, for example, aquaporins, which belong to the family of water channels. They enable the transport of water and of small molecules, such as glycerol, through biological channels and so far, 13 classes of aquaporins could have been identified in vertebrates. The classic aquaporin channels 1, 2, 4 and 5 are only permeable to water. The aquaporin channels 3, 7, 9, and 10 are also called aquaglycerolporins since they can also transport glycerol. This mini review discusses the immunohistochemical research on aquaporins, their range of applications, and respective forensic importance, their current limitations, and possible further implementations in the future.

## Introduction

Aquaporins (AQPs) belong to the family of water channels and enable the transport of water and small molecules, such as glycerol, through biological channels in many epithelial and endothelial cells (1–4). So far, 13 classes of AQPs have been identified in vertebrates. The classic AQPs (AQP1, 2, 4 and 5) are only permeable to water. The AQP channels 3, 7, 9, and 10 are also called aquaglycerolporins since they can also transport glycerol (5). AQP1 is located around the dermal capillaries (6). AQP3 is expressed in epidermal keratinocytes (7); the stratum corneum of the epidermis does not contain keratinocytes and AQP3-channels (8). AQP1, AQP4, and AQP9 are the best described AQPs in the brain (9), with AQP4 being the main water channel. They have a significant role in water homeostasis and neural signal transduction in the brain (10, 11), and their expression is rapidly induced by several stimuli, such as osmolarity (12, 13), mechanical, or chemical stress (14–16). In lung tissue, AQP5 represents the major water channels (17, 18). Although AQP5 expression appears to be induced by hypertonic stress (19) and suppressed by freshwater drowning (20) in murine lungs, its immunohistochemical expression patterns remain inconclusive in human lungs (20). In terms of forensic significance, AQP1 and AQP3 have been the most intensively researched AQPs in human skin to date. AQP1 is localized in fibroblasts, capillaries, and Langerhans and dendritic cells (21, 22). AQP3 is found in hypodermal adipocytes, dermal fibroblasts, epidermal keratinocytes, melanocytes, and dendritic and Langerhans cells and capillaries (21, 23–26). In particular, AQP3 has often been a central focus of forensic research in the past, as it appears to have an overriding function in skin hydration, epidermal barrier repair, and wound healing (26–31). AQP5, 7, 9, and 10 are also found in the skin and perform important functions but have not been systematically investigated forensically. In this mini review, we discuss the immunohistochemical research on aquaporins, their range of applications and respective forensic importance, their current limitations, and possible further implementations in the future.

## Methods

We reviewed the Medline dataset for studies published between 2009 and September 15, 2021 for AQPs in forensic context based on the updated 2020 PRISMA guidelines (Preferred Reporting Items for Systematic Reviews and Meta-Analysis) (32) for methodology and reporting. The words “aquaporin” and “forensic” were used to identify studies examining AQPs in forensic context. The following combination of Medical Subject Heading terms and Boolean operators was applied in our Medline search: “aquaporin” AND “forensic.” The reference lists of the included articles were manually searched for further studies. Two authors (J.P. and B.M.) conducted eligibility assessment and data extraction, assessment, and management independently. Only original research articles on human specimens published in English or German were considered for review. Eligibility of the article was determined by screening of titles and abstracts.

## Results

The initial search identified 49 studies, and after screening of the title, 23 studies remained for further inspection. After reviewing the abstracts, five studies were excluded because four were studies on animals and one did not present sufficient data for further analysis. Through manual searching of the reference lists, no further articles that matched the criteria could have been detected. The search eventually identified a total of 18 eligible studies between 2009 and 2021. Considering the study period, an average of 1.4 studies on AQPs in a forensic context have been published per year. Most studies were published in 2014, 2018, and 2021 (*n* = 3 each); moreover, almost half (44%) of the total identified studies were published between 2018 and 2021. The majority of studies addressed skin injuries and drowning (*n* = 5 each), followed by sudden infant death syndrome (SIDS) (*n* = 4). The remaining four studies covered different topics (pulmonary injury, traumatic brain injury, intoxication, discriminating between smothering and choking from sudden cardiac death). AQP4 was the most frequent subject of the studies (*n* = 6), followed by AQP1 (*n* = 5), AQP3, and AQP5 (*n* = 4 each). It was also striking that more than half of the studies (*n* = 11) on AQP were published in the I*nternational Journal of Legal Medicine*. Fourteen of the included studies were two-group designs, and five were multigroup designs. All studies combined had a total of 1,119 study specimens and 1,093 control specimens. An overview of the identified aquaporins and their respective potential applications is shown in above Table 1.

**Table 1:** Overview of aquaporins and their respective potential applications.

**Aquaporin**	**Potential field of application**
1	Pulmonary injuries, skin injuries, wound age, and vitality
2	Drowning
3	Burn injuries, skin injuries, wound age, and vitality
4	SIDS, intoxication, trauma, drowning
5	Drowning, smothering, chocking, sudden cardiac death
9	SIDS

## Discussion

### SIDS

The four studies that focused on SIDS investigated gene variations mainly by genetic analysis. AQP4 was predominantly studied. A decrease in AQP4 expression was observed in infants >12 weeks old. AQP4 expression was lower in infants and children with the rs2075575 CT/TT genotype than in those with the CC genotype (33). No differences in allele frequencies of the three AQP4 single-nucleotide polymorphisms (SNPs) previously shown to be associated with SIDS in Norwegian infants (rs2075575), severe brain edema (rs9951307), and increased brain water permeability (rs3906956) have been found between SIDS children and adult controls (34). Regarding the AQP1 gene, a significant association was found between the rs17159702 CC/CT and SIDS genotypes (*p* = 0.02). In the AQP9 gene, the combination of a TT genotype of rs8042354, rs2292711, and rs13329178 was more common in SIDS cases than in controls (*p* = 0.03). In the SIDS group, an association was found between genetic variations in the AQP1 gene and maternal smoking and between the 3×TT combination in the AQP9 gene and the finding of lifeless infants in the prone position (35). For AQP4, one study found an association between the T allele and CT/TT genotypes of rs2075575 and SIDS (C vs. T, *p* = 0.01; CC vs. CT/TT, *p* = 0.03), but none for the other three SNPs. For the SNP = rs2075575, an association between the brain/body-weight ratio and genotype was also found in SIDS patients at 0.3–12 weeks of age (*p* = 0.014, median ratio of CC = 10.6, CT/TT = 12.1) (36).

We concluded that specific variations in the genes of AQP1, 4, and 9, along with external risk factors and probably other genetic factors, represent a genetic predisposition that make an infant vulnerable to sudden death. Furthermore, the AQP4 CT/TT genotypes appear to be associated with an increased brain-to-body-weight ratio in infants (35, 36). Additionally, AQP4 expression in infants may be influenced by both age and genotype, but the role of AQP4 in the pathogenesis of SIDS remains to be elucidated (33). Yet another study concluded that variations in the AQP4 gene were of limited importance as predisposing factors in Caucasian SIDS children (34).

### Drowning

Five studies have examined AQP expression in drowning by immunohistochemistry and gene expression analysis. In addition, studies focused on the distinction between freshwater drowning (FWD) and saltwater drowning (SWD). Intrapulmonary gene expression of AQP5 was significantly decreased in FWD relative to that in SWD and other cases, which may be due to suppressed AQP5 expression in type I alveolar epithelial cells by hypotonic water to prevent hemodilution from a physiological perspective (20). Another study found that there was no statistically significant difference in lung-tissue AQP5 between SWD, FWD, and controls (37). In the kidneys, there were no significant differences in the expression of AQP1 and AQP4 between the FWD, SWD, and control groups. Immunohistochemically, AQP2 was predominantly expressed in the apical plasma membrane of collecting duct principal cells in all kidney samples from FWD and SWD. Morphometrically, there was significantly increased AQP2 expression in the apical plasma membrane of collecting ducts in the SWD group compared with the FWD and control groups (38). Brain samples showed that the mean value of AQP4-positive astrocytes was significantly higher in the FWD group than in the SWD and control groups. In addition, AQP4 expression was significantly lower in the SWD group than in the control group (*p* < 0.05) (39). For AQP2 (as well as arginine vasopressin), there was stronger statistically significant expression in renal tissue in the SWD group (*p* < 0.05) than in the FWD and control groups (40). The authors of the respective studies concluded that immunohistochemical detection of AQP2 in the kidney and AQP4 detection in the brain could be valuable markers for differentiating between FWD and SWD. Two studies of AQP5 expression in lung tissue yielded conflicting results.

### Skin Injuries

Five studies investigated AQP expression in different types of skin lesions, mainly by immunohistochemistry. In the central portions of burn wounds where the epidermis and dermis are destroyed, no AQP3 was found in one study, but strong AQP3 staining was detected along the edge of the burn wound. Western blot analysis also showed stronger staining along the burn wound than in unburned control skin. Quantification showed significantly more AQP3 along the burn wound than in unburned skin and no AQP3 expression in the center of the burn wound (41). Examination of the expression of AQP1 and AQP3 in skin samples of the neck in cases of neck compression showed no significant difference in the AQP1 expressions in dermal capillaries between the study and control groups. In contrast, weak positive signals for AQP3 were detected in uninjured skin samples, and the positive signals again appeared more intense in keratinocytes in the compression regions. Morphometric analysis revealed that the proportion of AQP3-expressing keratinocytes was significantly increased in the neck compression regions relative to that in the control groups (42). The same authors studied the expression of AQP1 and AQP3 in human skin wounds that were classified into different groups according to their post-infliction interval. In uninjured skin samples, AQP1 and AQP3 were detected in dermal vessels and keratinocytes, respectively, at low levels, and the percentage of AQP1-positive vessels and number of AQP3-positive keratinocytes appeared to increase with wound age (43).

Another study combined gene analysis and immunohistochemistry to assess the expression of AQP1 and AQP3 in the skin of forensic autopsy cases and its value in the differential diagnosis of antemortem and post-mortem burns. AQP3 gene expression was significantly higher in the skin of antemortem burn cases than in post-mortem burn cases, mechanical wounds, and control cases. In contrast, immunohistochemical evaluation showed no differences in AQP3 expression patterns between control, antemortem, and post-mortem burn skin. This finding was attributed to a probable increase in dermal AQP3 gene expression to maintain water homeostasis in response to dehydration caused by the burn (44). In another study, the expression of AQP1 and AQP3 was investigated in various skin injuries caused by blunt, sharp, and thermal force trauma; strangulation marks; gunshot wounds; and frostbite. In another study, the expression of AQP1 and AQP3 was investigated in various skin injuries caused by blunt, sharp, and thermal force trauma; strangulation marks; gunshot wounds; and frostbite. There was no correlation between AQP3 expression and age, sex, body mass index, duration of agony, and post-mortem interval. For AQP1, there were no differences between injured and uninjured skin (45).

To summarize the conclusions of these five studies, immunohistochemical detection of AQP3 in neck skin could be valuable as a forensic marker for the diagnosis of antemortem compression or as a vital signs marker. Furthermore, immunohistochemical analyses of AQP1 and AQP3 in human skin wounds seem to be capable of supporting the objective accuracy of wound-age determination and determination of AQP3 gene expression seems to be useful for the forensic molecular diagnosis of antemortem burn wounds.

### Other Research Areas

Four other studies investigated different aspects of AQP expression. One study compared intrapulmonary expressions of AQP1 and AQP5 via mRNA quantification as markers of water homeostasis between cases with smothering and choking or strangulation and with sudden cardiac death and acute brain injury. AQP5, but not AQP1, showed suppressed expression in smothering compared with expression in strangulation and sudden cardiac death and death from acute brain injury (46). Furthermore, molecular pathological analysis of post-traumatic alveolar injury and systemic responses affecting pulmonary edema, including AQP1 and APQ5 mRNA, expression of AQP1 in lung tissue was significantly higher in subacute sharp force injury than in the other groups. Regarding AQP5 mRNA expression, there were no differences among all groups. On immunohistochemical examination, AQP1 was clearly detectable in all vascular endothelial cells but showed no differences in distribution and intensity. AQP5 was weakly detectable in a linear pattern in type-1 alveolar epithelial cells and sporadically in interstitial macrophages, as shown in the other study by this group (47). A study on post-mortem brain mRNA and immunohistochemical expressions, including AQP4, in forensic autopsy cases of carbon monoxide methamphetamine and phenobarbital intoxications compared with different cases of traumatic injury showed higher expression of AQP4 in methamphetamine intoxications. Immunostaining results showed substantial interindividual differences between groups, with no apparent differences in distribution or intensity between all causes of death (48). Another study examined expression of AQP4 and correlation with hypoxia and neuroinflammation in human traumatic brain injury. AQP4 showed a significant and progressive increase between the control group and groups 2 (one-day survival) and 3 (3-day survival) from the acute stages of traumatic insult. In addition, there was an increase in AQP4 immunopositivity in groups 4 (7-day survival), 5 (14-day survival), and 6 (30-day survival), which may indicate upregulation of AQP4 at 7–30 days relative to that on day 1 (49).

In summary, mRNA quantification of AQP5 could distinguish smothering from choking and sudden cardiac death. Systematic analysis of gene expression, including of AQP4, via real-time polymerase chain reaction could be a useful procedure in forensic death investigations of methamphetamine intoxications since AQP4 might be upregulated in the brain during this kind of intoxication. Furthermore, AQP4 might be useful for estimating the time of survival in traumatic brain injuries.

## Conclusions

In the 13-year period studied, relatively few studies were published that addressed the forensic significance of AQPs. The main focus of these studies was on SIDS, skin injuries, and drowning, in particular, on the distinction between FWD and SWD. The studies on SIDS mainly involved gene analysis, whereas the other two main topics involved either immunohistochemistry or a combination of the two. Specific gene variations of AQP1, 4, and 9, in combination with other influencing factors, might make infants more susceptible to onset of SIDS, although the importance of AQPs, especially AQP4, remains largely unclear. In drowning, AQP2 in the kidney and APQ4 in the brain appear to be useful with respect to distinguishing FWD from SWD, although such a distinction will probably only have relevant applications at a few forensic institutions with appropriate geographic settings. For skin injuries, AQP3 in particular seems to be a possible complementary test; e.g., to detect the vitality of (burn) wounds or antemortal skin compressions and to narrow the wound age. In addition, AQP5 could be used to distinguish smothering from sudden cardiac death, and AQP4 could be used to temporally delineate survived traumatic brain injury. Interesting research approaches could be found in the respective studies indicating that investigation of AQPs potentially can provide considerable added value for answering some questions. A combination of immunohistochemistry and gene expression analysis appears to be useful in each case to increase statistical significance. An overview of the complementary biomarkers is shown in Table 2.

**Table 2:** Overview of fields of application and the respective applicable aquaporins as well as supplementary biomarkers.

**Potential field of application**	**Aquaporin**	**Supplementary biomarkers**
Skin injuries, wound age, and vitality	1, 3	MMP1, MMP9 (50), EPC (51), Tryptase, IL-15, CD15 (52), VEGF (53), Ubiquitin (54), IL-1α (55), IL-8, MCP-1, MCP-1α (56)
Drowning	1, 2, 4, 5	SP-A (37), AVP (40)
Burn injuries	3,	HIF (57)
Trauma	1, 4, 5	MMP2, MMP9, ICAM-1, Claudin-5 (47), GFAP, HIF-1α, IBA-1, CD68 (49), VEGF (16)
Smothering/chocking	5	Claudin-5 (46)
Intoxication	4	MMP2, MMP9, Claudin-5 (48)
Sudden cardiac death	5	
SIDS	1, 4, 9	

*MMP, metalloproteinase; VEGF, vascular endothelial growth factors; EPC, endothelial progenitor cells; IL-15, Interleukin-15; SP-A, Surfactant protein A; AVP, Hormone Arginine Vasopressin; HIF, Hypoxia-inducible Factors; GFAP, Glial fibrillary acidic protein; IBA, Ionized calcium-binding adaptor molecule; ICAM, Intercellular adhesion molecule*.

This mini-review was limited by the fact that only studies on human material were included. Experimental studies or animal studies were deliberately omitted. Emphasis was placed on studies where immediate practical application is possible and where the results may, at best, add value to criminal investigations or court proceedings.

However, it is striking that there are only a few, if any, follow-up studies to the respective studies and that these were often conducted by the same research group. It must be noted, therefore, that the authors' frequent claims that further research is needed to evaluate the value and applicability of AQPs in the forensic context have gone largely unheeded by the scientific community.

Nevertheless, this mini review certainly shows the potential of AQPs in forensics and, despite the relatively few studies that have been conducted on human specimen to date, that there are very interesting and potentially relevant research approaches worth pursuing.

## Author Contributions

JP: conception and design of study and drafting the manuscript. JP and BM: acquisition of data. JP, BM, and ED: analysis and/or interpretation of data, revising the manuscript critically for important intellectual content, and approval of the version of the manuscript to be published. All authors contributed to the article and approved the submitted version.

## Conflict of Interest

The authors declare that the research was conducted in the absence of any commercial or financial relationships that could be construed as a potential conflict of interest.

## Publisher's Note

All claims expressed in this article are solely those of the authors and do not necessarily represent those of their affiliated organizations, or those of the publisher, the editors and the reviewers. Any product that may be evaluated in this article, or claim that may be made by its manufacturer, is not guaranteed or endorsed by the publisher.
